# Sociodemographic factors and career growth as determinants of staff retention among nurses in a military hospital in Saudi Arabia

**DOI:** 10.1097/MD.0000000000050431

**Published:** 2026-08-28

**Authors:** Amal Bany Ateah, Ahmad Aboshaiqah, Ahmad M. Rayani

**Affiliations:** a Nursing Department, Head Nurse, King Salman Armed Forces Hospital, Tabuk, Saudi Arabia; b Administration and Education Department, College of Nursing, King Saud University, Riyadh, Saudi Arabia; c Community and Psychiatric Mental Health Nursing Department, College of Nursing, King Saud University, Riyadh, Saudi Arabia.

**Keywords:** career growth, military hospital, nurses, Saudi Arabia, sociodemographic factors, staff retention, turnover intention

## Abstract

Nurses are the backbone of healthcare systems, and their retention remains a critical global challenge, as high attrition leads to a loss of expertise, continuity of care, and patient trust. The main objective of this study is to examine whether sociodemographic factors and career growth act as determinants of staff retention among nurses working in a military hospital in Saudi Arabia. A cross-sectional analytic study was conducted among nurses employed at an Armed Forces Hospital in Saudi Arabia over a 3-month period between June and August 2025. Data were collected using an online self-administered questionnaire distributed via social media platforms. The questionnaire included sections on sociodemographic characteristics, career growth, and turnover intention. Statistical Package for Social Science (SPSS) version 27 was used to analyze the data. A total of 350 nurses completed the questionnaire, yielding a response rate of 92.1%. Most nurses reported positive perceptions of career growth, with 78.3% indicating improved emotional control and 48% strongly agreeing that their professional competence had improved. Over half of the participants (51.7%) strongly agreed that their professional prestige had increased due to work achievements, and many perceived timely promotions according to organizational ladders. Turnover intention was low, as 66.9% strongly disagreed with having considered leaving their job, and 75.4% strongly disagreed with frequently thinking about seeking alternative employment. The mean career growth score was 66.71 ± 19.78, while the mean turnover intention score was 10.83 ± 3.92. A strong negative correlation was found between career growth and turnover intention (*r* = −0.718, *P* < .001). Nurses in this military hospital demonstrated positive perceptions of career growth and low turnover intention, reflecting high job satisfaction and organizational commitment. Enhancing career development opportunities may play a crucial role in sustaining nurse retention within military healthcare settings.

## 1. Introduction

Nurses constitute the backbone of the global healthcare system, and their retention is a pivotal concern for healthcare systems worldwide, including Saudi Arabia, where attrition results in the loss of competence, consistency, and patient trust.^[1,2]^ Nurse retention is driven by rising care demands, workforce shortages, and substantial turnover and recruitment expenses.^[1]^ Nurses’ turnover intention correlates with diminished patient care quality, heightened operational burdens, and lowered organizational performance, highlighting the necessity of finding determinants of retention and satisfaction within nursing workforces.^[1,3]^ However, research has indicated that retention refers to an organization or system’s capacity to retain its workers, clients, or participants over time. Because it directly affects stability, performance, and growth, it is an essential indicator in human resources education and business.^[4]^

Although skill development, professional promotion, and job extension are known as career growth, and they have become a crucial organizational aspect affecting nurses’ work experiences.^[5]^ According to recent studies, positive workplace outcomes like job satisfaction and organizational loyalty are strongly linked to views of career progression.^[5]^ As an illustration of the importance of career advancement pathways in maintaining a stable workforce, research has demonstrated that nurses who perceive organizational support for career growth tend to report better engagement and lower turnover intention.^[5]^ In addition opportunities for career advancement are widely acknowledged as a critical factor in determining nurses’ job satisfaction and long-term loyalty to their employers.^[6]^ Nurses are empowered by professional development, clear career trajectories, and possibilities for growth. These factors also enhance clinical abilities and have a positive impact on retention results.^[6]^ Nurses’ intentions to quit their existing jobs have been connected to inadequate career advancement, highlighting the significance of organized career development as a component of workforce sustainability measures.^[7]^ While nursing staff retention is influenced by various organizational and human factors such as job satisfaction, leadership styles, and work environment, evidence from the literature suggests that retention intentions are associated with moderate levels of job satisfaction and are further shaped by sociodemographic characteristics, including age, years of experience, and nationality.^[3,8]^

House et al investigated the relationship between professional roles, demographic characteristics, job satisfaction, and retention among nurses in a military medical institution. Their findings indicated that professional role and demographic variables were not significantly associated with job satisfaction; however, among military personnel, professional role, race/ethnicity, and educational level were significantly related to intention to stay.^[9]^ In line with these findings, a systematic review by Marufu et al identified leadership and management practices, education and career development opportunities, staffing levels, organizational culture, and remuneration as key determinants of nurse retention in hospital settings.^[10]^ Monthly income also plays a critical role in shaping job satisfaction and retention. Beyond representing the financial value of nursing work, compensation interacts with sociodemographic characteristics to influence nurses’ career decisions. Previous studies have demonstrated strong associations between salary levels, work experience, and job satisfaction, which in turn affect nurses’ intentions to remain in or leave their positions.^[11]^ Furthermore, demographic factors such as age, education, and professional experience have been shown to significantly influence job satisfaction, organizational commitment, and intention to stay among clinical nurses.^[8]^ Understanding these sociodemographic influences is particularly important in Saudi Arabia, where the nursing workforce is culturally diverse, and retention strategies must be tailored to varying needs and career expectations.^[8]^ Given the interrelated nature of career growth opportunities, staff retention, compensation, and sociodemographic characteristics, a comprehensive examination of these factors among nurses is essential to inform effective workforce retention strategies.

Career growth has been recognized as a multidimensional organizational construct encompassing professional ability development, career goal progress, promotion opportunities, and remuneration growth. Previous studies suggest that employees who perceive greater opportunities for career growth are more likely to demonstrate organizational commitment and lower turnover intention. Conversely, limited career advancement opportunities may increase employees’ intention to leave their organization. Although demographic characteristics such as age, education, years of experience, and professional position may influence perceptions of career growth, these effects are often indirect and mediated by organizational policies, career development opportunities, and workplace support. Based on this theoretical framework, the present study examined the relationship between career growth and turnover intention while considering relevant demographic characteristics as potential covariates. The main objective of this study is to examine whether sociodemographic factors and career growth act as determinants of staff retention among nurses working in a military hospital in Saudi Arabia.

## 2. Materials and methods

A cross-sectional analytical study was conducted among nurses working in a military hospital in Saudi Arabia over a period of 3 months from June to August 2025 using social media platforms (WhatsApp). The study sample included nurses who were currently registered with the Saudi Commission for Health Specialties, employed at the study site, and willing to participate. Eligible participants were those who were able to read and complete the online questionnaire independently and who provided informed consent to participate in the study. Nurse interns and nurses working in other hospitals or other regions of Saudi Arabia were excluded from the study. Ethical approval from the Human Ethics Committee, King Saud University (KSU; KSU-HE-25757) was obtained before data collection. Additionally, the study adhered to the principles of the Declaration of Helsinki and best clinical practices throughout the investigation.

### 2.1. Sample size and study population

This study employed convenience sampling to recruit nurses from various departments of a military hospital. The estimated total population consisted of 9870 nurses currently working at the study site. The required sample size was calculated using the formula for estimating a proportion in a finite population.

The sample size was calculated using the following formula:


$$Sample\;size=\left[z^{2}\times p\left(1-p\right)\right]/e^{2}/1+\left[z^{2}*p\left(1-p\right)\right]/e^{2}*N]$$

$$\begin{array}{c} Z\text{-}score=The\;Z\text{-}value\;for\;a\;95\%\;conf\;idence\;level,\;which\;is\;z=1.96 \end{array}$$

$$\begin{array}{c} Margin\;of\;error=0.05\;\left(5\%\right) \end{array}$$

$$\begin{array}{c} Standard\;of\;deviation=p=0.5 \end{array}$$

$$\begin{array}{c} N=Nurses\;population=9870 \end{array}$$

$$\begin{array}{c} \left(0.5-1\right)\times 0.5\times 21.96=sample\;size \end{array}$$

$$\begin{array}{c} \left(\left(1-0.5\right)\times 0.5\times 21.96\right)+20.0 \end{array}$$

$$\begin{array}{c} 9870\times 20.05 \end{array}$$

$$\begin{array}{c} 2z\times p\times \left(1-p\right)=0.9604 \end{array}$$

A total of 370 nurses were required for the study. To account for potential nonresponse or incomplete questionnaires,^[12]^ the minimum required sample size was adjusted using a nonresponse correction formula. A 3% nonresponse rate was assumed because the study involved institutional-based data collection among healthcare professionals with direct distribution and follow-up of questionnaires, which was expected to yield a high response rate. Accordingly, the adjusted sample size was calculated as 381 participants and rounded to 380 for feasibility and ease of reporting.

### 2.2. Study questionnaires

The questionnaire used in this study was adopted and comprised 3 sections, with a total of 30 items addressing job satisfaction and Turnover Intention.^[13,14]^ Section 1 focused on the demographics of the nurses, including age, gender, nationality (Saudi/Filipino/South Asians like Indians, Pakistanis, and Bangladeshi), others (Egypt, Yemen, Sudan), years of nursing experience, current department, level of education, and monthly income. Section 2 consisted of questions about the career growth scale, which assessed nurses’ perceptions of professional ability and attribute improvement (7-items), career promotion and prestige (6–items), and progress toward career goals (4–items). The third section assessed nurses’ intention to leave their current job and consisted of 6 items. Responses were recorded using a Likert-type scale ranging from strong disagreement to strong agreement, with higher scores indicating greater perceived career growth or stronger turnover intention. Following the initial draft, the questionnaire underwent content validation by a team of 3 independent evaluators (one researcher and 2 professors from the department of nursing administration and education). Subsequently, the questionnaires were pilot-tested among randomly selected nurses (n = 30) to assess language flow and readability. The internal consistency of the 17-item Career Growth Scale was assessed using multiple reliability indices. The scale demonstrated excellent internal consistency, with a Cronbach’s α coefficient of 0.983. Similarly, McDonald’s omega coefficient was 0.982, further confirming the excellent reliability of the instrument. Item-level analysis showed that the corrected item-total correlations ranged from 0.774 to 0.961, exceeding the recommended threshold of 0.30 and indicating that all items contributed meaningfully to the overall construct. The “Cronbach’s α if item deleted” values ranged from 0.981 to 0.983, demonstrating that the removal of any individual item did not improve the internal consistency of the scale. Therefore, all 17 items were retained for the final analysis. The average item scores ranged from 3.75 ± 1.40 to 4.06 ± 1.25, indicating a satisfactory distribution of responses across the scale items. The intraclass correlation coefficient (ICC) further supported the reliability of the instrument. The single-measure ICC was 0.771 (95% CI: 0.744–0.799), indicating good agreement among individual items, whereas the average-measure ICC was 0.983 (95% CI: 0.980–0.985; *P* < .001), demonstrating excellent reliability of the overall scale.

The 6-item Staff Retention Scale demonstrated acceptable internal consistency, with a Cronbach’s α coefficient of 0.75 and a standardized Cronbach’s α of 0.76. McDonald’s omega was 0.76, supporting the reliability of the instrument. Corrected item-total correlations ranged from 0.41 to 0.58, indicating that all items contributed adequately to the overall construct. Cronbach’s α if item deleted ranged from 0.68 to 0.72, suggesting that the removal of any individual item did not improve the internal consistency of the scale. The average-measures intraclass correlation coefficient was 0.75 (95% CI: 0.68–0.81), indicating acceptable reliability. Therefore, all 6 items were retained for the final analysis.

### 2.3. Exposure and outcome variables

In this study, the outcome variable was staff retention, operationalized as nurses’ turnover intention, which reflects their likelihood of leaving their current job. The exposure variables included career growth, encompassing professional ability and attribute improvement, career promotion and prestige, and progress toward career goals, as well as sociodemographic characteristics, including age, gender, nationality, level of education, years of nursing experience, department, and monthly income.

### 2.4. Data analysis

Statistical Package for Social Science (SPSS, IBM corporation) version 27 was used to analyze the data. Categorical variables were summarized using descriptive statistics, such as frequencies and percentages, while continuous variables were assessed using means and standard deviations. The Shapiro–Wilk test was used to check for normality, and it was discovered that all of the data were normally distributed. In order to ascertain the statistical differences between variables, parametric tests such as the *t*-test for variables with 2 categories and ANOVA for more than 2 categorical variables were utilized. Relationships between study factors, turnover intention, and career growth were examined using correlation analysis. The association between career growth and turnover intention was evaluated using Pearson’s correlation coefficient, while the relationship between career growth and sociodemographic variables was evaluated using Spearman’s correlation coefficient. A *P*-value of less than .05 was required for every test employed in this investigation to be deemed statistically significant.

**Figure:**
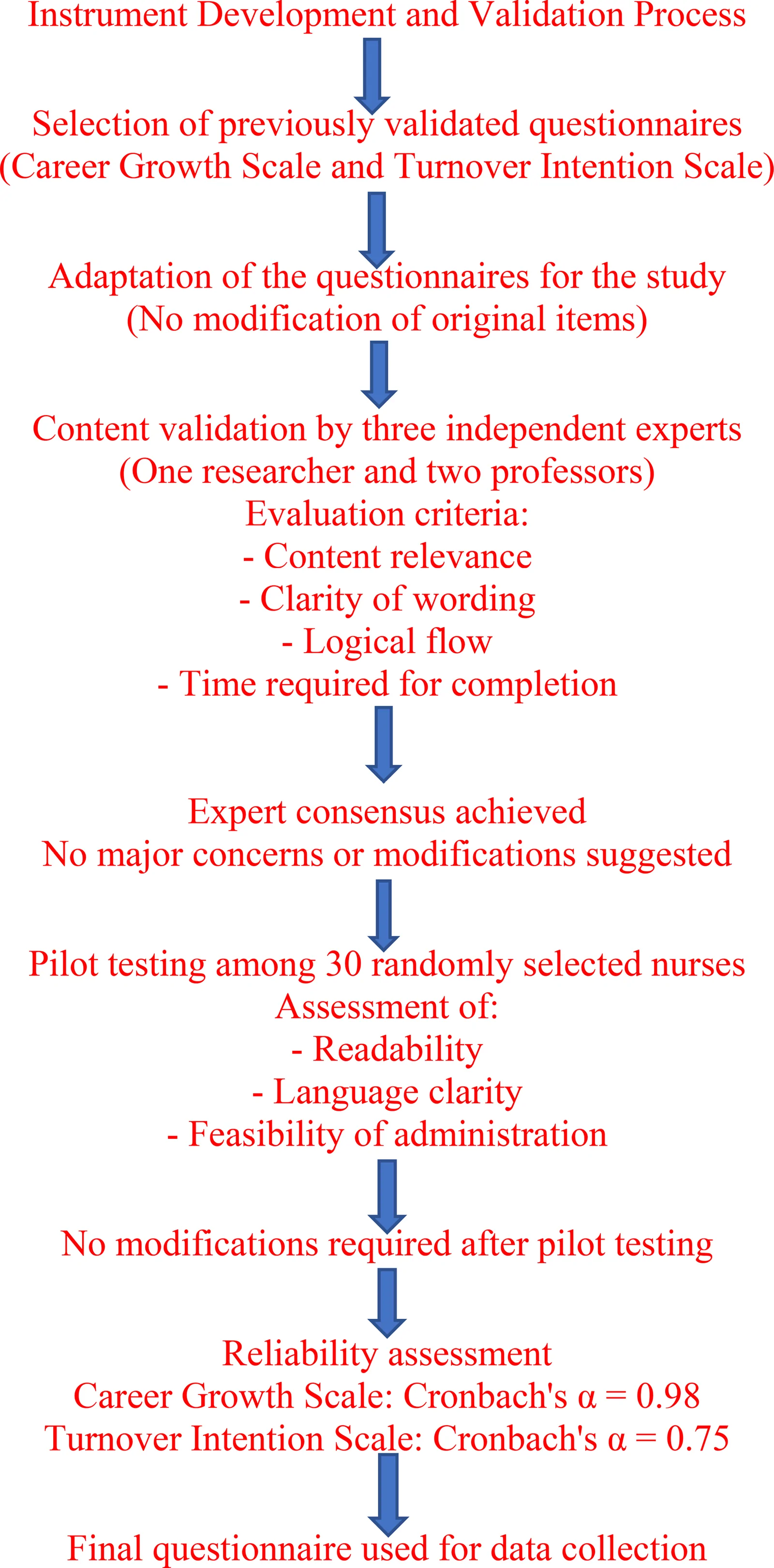


## 3. Results

This section presents the study findings, focusing on how sociodemographic factors and career growth are associated with staff retention, as reflected by turnover intention, among nurses working in a military hospital in Saudi Arabia. A total of 350 nurses completed the questionnaires, yielding a response rate of 92.1%. Table 1 presents the demographic characteristics of nurses working in the military hospital. The majority of participants were aged between 31 and 40 years (57.4%) and were predominantly female (89.7%). In terms of nationality, Saudi nurses represented the largest group (33.7%), followed by Filipino nurses (25.7%) and other Asian nationalities (31.4%), reflecting the multicultural composition of the nursing workforce. Regarding professional experience, nearly 56.0% of nurses reported more than 10 years of experience, with 28.9% having over 15 years of nursing experience, indicating a highly experienced workforce. Nurses were employed across various departments, with the largest proportions working in outpatient services (19.1%), medical/surgical units (14.3%), and intensive care units (10.9%). Approximately one-third of the participants (33.7%) were categorized under other departments, reflecting broad clinical representation. Most nurses held a bachelor’s degree in nursing (62.6%), followed by diploma holders (32.3%), while a small proportion had postgraduate qualifications. Regionally, the majority of nurses were from the northwestern region (72.3%), with smaller proportions representing other regions. In terms of income, more than half of the participants (58.9%) reported a monthly income between 5000 and 9999 Saudi Arabian Riyals, whereas only 3.4% earned 20,000 Saudi Arabian Riyals or more.

**Table 1:** Demographics characteristics of nurses working in military hospital.

Variables	Frequency (n)	Percentage (%)
Age		
Between 25 and 30	65	18.6
31–40	201	57.4
41–50	67	19.1
>50 yr	17	4.9
Gender		
Male	36	10.3
Female	314	89.7
Nationality		
Saudi	118	33.7
Filipino	90	25.7
South Asians	110	31.4
Others	32	9.1
Years of experience in nursing		
<1 yr	4	1.1
1–5 yr	65	18.6
6–10 yr	95	27.1
11–15 yr	85	24.3
More than 15 yr	101	28.9
Current department		
Nursing administration	23	6.6
Emergency	22	6.3
Intensive care unit (ICU)	38	10.9
Medical/surgical	50	14.3
Outpatient	67	19.1
Paediatrics	12	3.4
Maternity	9	2.6
Oncology	11	3.1
Other	118	33.7
Level of education		
Diploma in Nursing	113	32.3
Bachelor’s Degree in Nursing	219	62.6
Master’s Degree in Nursing	16	4.6
PhD in Nursing	2	.6
Monthly income		
Less than 5000 SAR	15	4.3
5000–9999 SAR	206	58.9
10,000–14,999 SAR	74	21.1
15,000–19,999 SAR	43	12.3
20,000 SAR or more	12	3.4

ICU = Intensive care unit, SAR= Saudi Arabian Riyals.

### 3.1. Professional ability and attribute improvement

**Figure 1.**
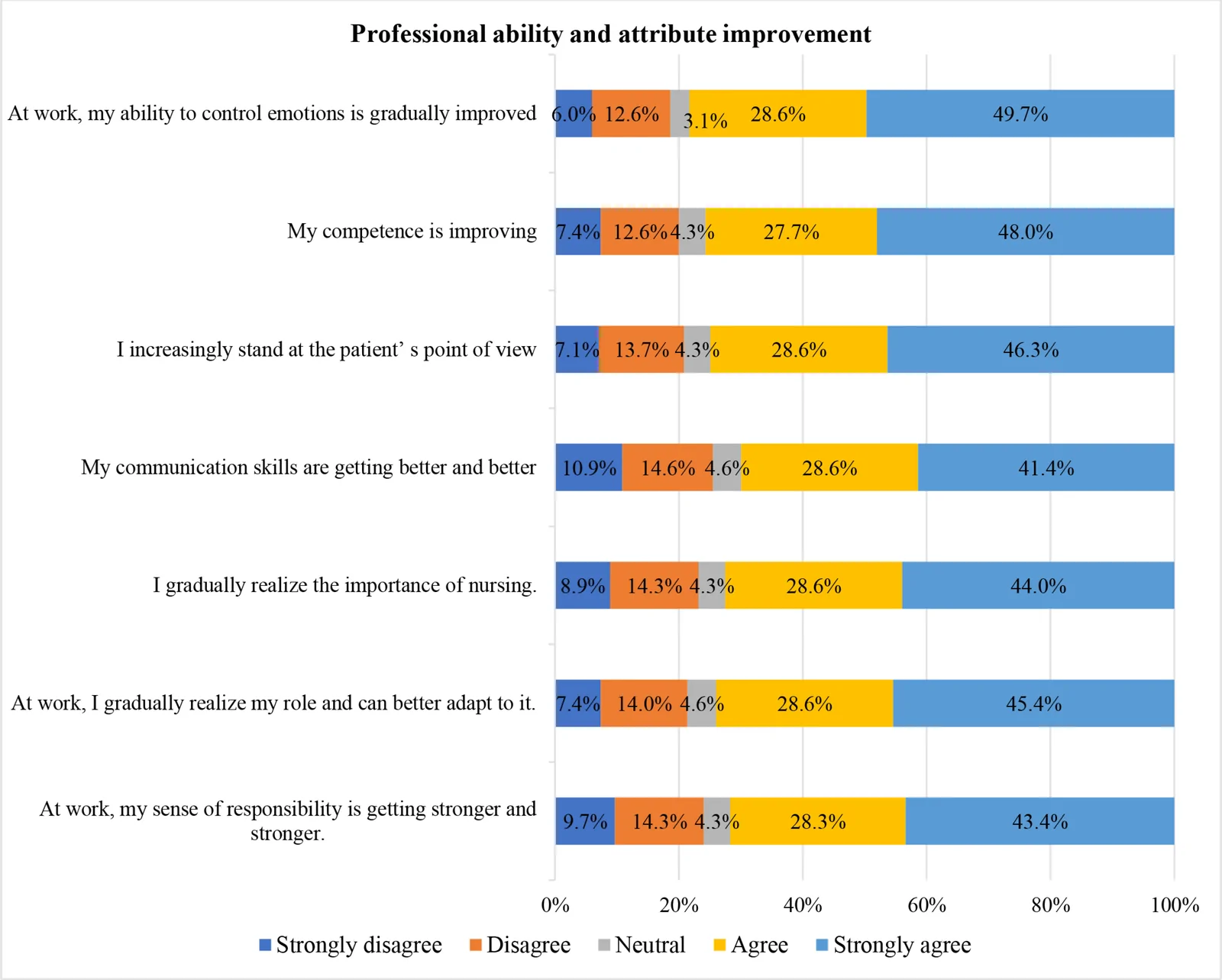
Nurses responses on professional ability and attribute improvement.

Figure 1 presents nurses’ responses regarding their professional abilities and personal attributes. Overall, the findings show a positive pattern across all items. A substantial proportion of nurses (78.3%) either agreed (28.6%) or strongly agreed (49.7%) that their ability to control emotions had improved over time. Similarly, 48.0% of participants strongly agreed that their professional competence was improving. Improvements in communication skills were also evident, with 41.4% strongly agreeing that their skills were getting better. In addition, 46.3% of nurses strongly agreed that they were increasingly able to understand the patient’s perspective. Regarding personal and professional development, 43.4% strongly agreed that their sense of responsibility had become stronger, while 45.4% strongly agreed that they were better able to adapt to their professional roles.

### 3.2. Career promotion and prestige increase

**Figure 2.**
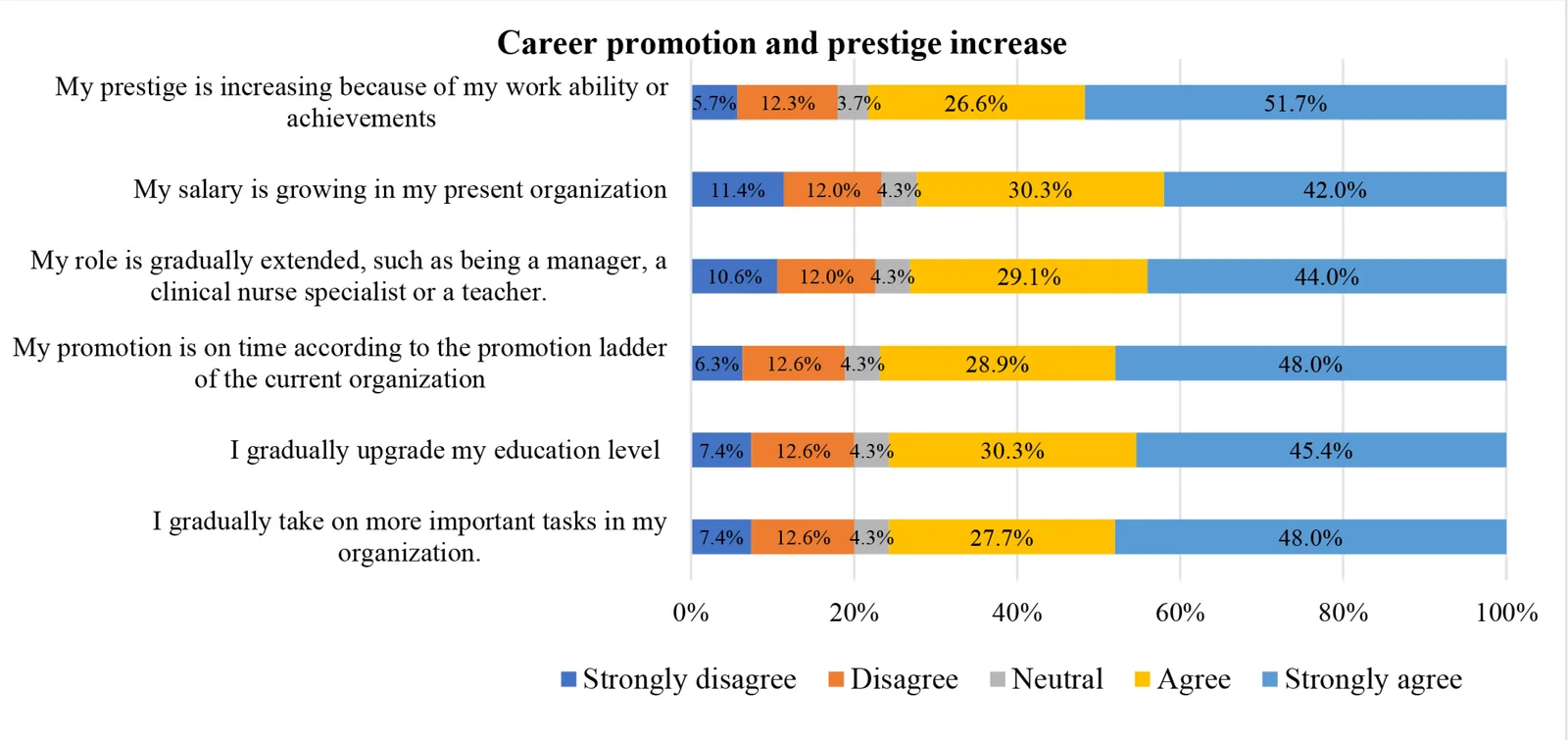
Nurses reprocess on career promotion and prestige increase.

The highest level of strong agreement was observed for the statement that prestige is increasing because of work ability or achievements (51.7%; Fig. 2), followed by “my promotion is on time according to the promotion ladder of the current organization” (48%), and salary growth within the current organization (42.0%), suggesting that nurses in the current study perceived positive progress in career promotion, salary growth, and professional recognition as shown in Figure 2.

### 3.3. Career goal progress

**Figure 3.**
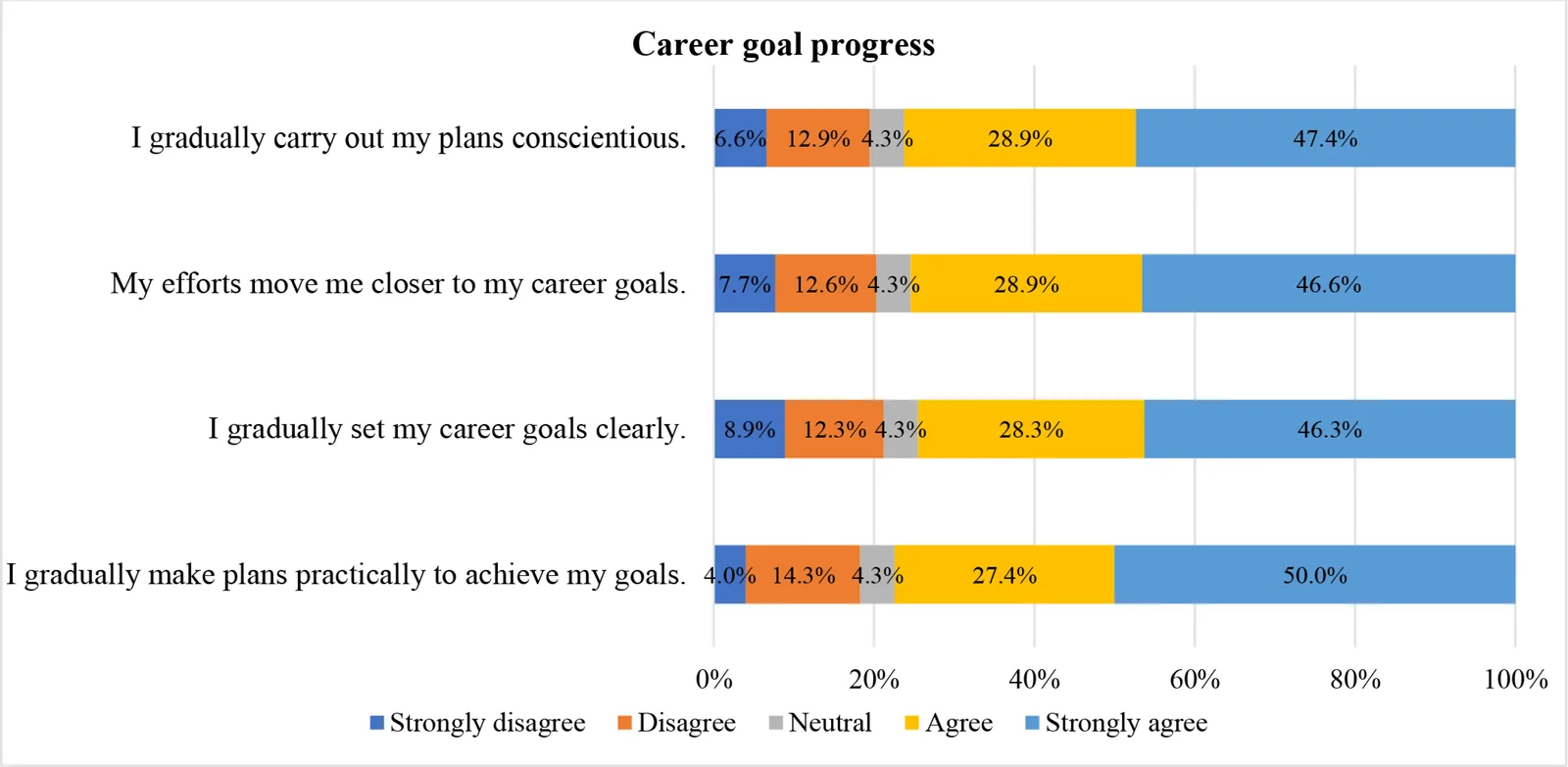
Nurses responses on career goal progress of career growth scale.

Approximately 46.3% to 50.0% of nurses strongly agreed that they were setting clear career goals, making practical plans, and moving closer to achieving those goals. An additional 27.4% to 28.9% agreed with these statements, indicating that most nurses felt confident about their career planning and believed they were making steady progress toward their professional goals. Figure 3 shows the nurses’ responses towards career goal progress.

### 3.4. Turnover intention

**Figure 4.**
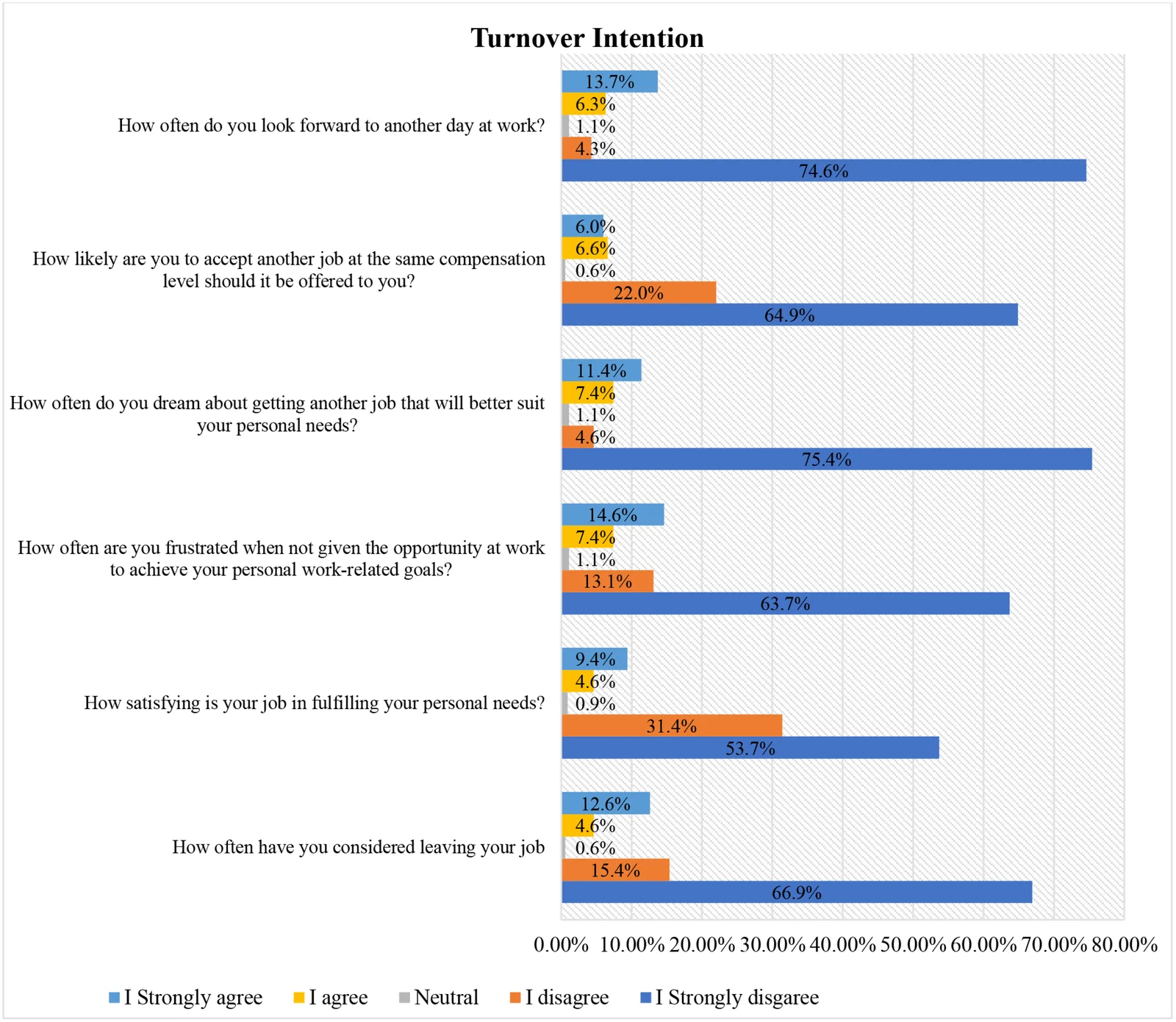
Nurses’ responses on turnover intention.

A significant number of nurses disagreed or strongly disagreed with statements regarding their departure from work (Fig. 4). For example, 66.9% strongly disagreed with the idea that they had considered leaving their job, while 75.4% strongly disagreed with the notion that they often thought about finding another job. Similarly, 74.6% strongly disagreed with the idea that they did not look forward to another day at work. Statements related to turnover had a relatively low level of agreement, ranging from 4.6% to 15.4%. Neutral responses accounted for 6.0% to 14.6%. The results indicate that the majority of nurses were satisfied with their current jobs and did not intend to leave their organization. The detailed responses were given in Figure 4.

The mean score for career growth among nurses was 66.71 ± 19.78 (range = 0–68), while the mean turnover intention score was 10.83 ± 3.92 (range = 0–20). Table 2 presents the correlation analysis between career growth and selected study variables. A strong and statistically significant negative correlation was observed between career growth and turnover intention (*r* = −0.718, *P* < .001). This indicates that nurses who reported higher levels of perceived career growth were substantially less likely to express intentions to leave their current positions. In contrast, no statistically significant correlations were found between career growth and the remaining demographic or work-related variables, including age, gender, nationality, years of nursing experience, current department, level of education, and monthly income (all *P* > .05). The correlation coefficients for these variables were close to 0, suggesting minimal or no linear association with perceived career growth.

**Table 2:** Correlation between career growth and study variables.

Variables	Correlation coefficient (*r*)	Statistical significance
Career growth and turnover intension	−.718*	<.001
Career growth and age	−.004	.937
Career growth and gender	.028	.598
Career growth and nationality	.031	.569
Career growth and years of experience	−.050	.354
Career growth and department	−.002	.969
Career growth and education	.001	.986
Career growth and monthly income	−.089	.096

* The correlation is statistically indicative at a significance level of 0.01. The relationship between career growth and turnover intension is presented by Pearson correlation. The relationship between career growth and other variables is presented by Spearman correlation.

The independent samples t-test was conducted to compare the mean scores of career growth by gender. Male participants reported a slightly higher mean score of career growth (M = 66.93, SD = 19.80) than female participants (M = 64.80, SD = 19.80). However, this difference was not statistically significant (*t* = −0.61, *P* = .541). The effect size was small (Cohen’s *d* = 0.11), indicating a negligible practical difference between males and females.

The results of ANOVA did not reveal any significant difference between the mean of career growth and nurse’s age, gender, education, department nationality and monthly income, suggesting that they did not affecting on the career growth of the nurses. On the other hand, the multiple linear regression analysis examined predictors of career growth among nurses. The overall model was statistically significant (*F* = 46.04, *P* < .001) and explained approximately 52% of the variance in career growth (*R*^2^ = .519; adjusted *R*^2^ = .508), indicating a strong model fit. Among the predictors included in the model were gender, age, nationality, level of education, years of nursing experience, current department, monthly income, and teamwork intensity. However, turnover intention emerged as a significant predictor of career growth. Specifically, turnover intention was negatively associated with career growth (*B* = −3.61, β = −.715, *P* < .001), suggesting that higher levels of turnover intention were linked to lower perceived career growth. All other demographic and professional variables were not statistically significant predictors (*P* > .05), indicating that they did not independently influence career growth in this sample (Table S1, Supplemental Digital Content 1).

Overall, the findings indicate that nurses reported generally positive perceptions of career growth across its different dimensions, including professional abilities, career advancement, and progress toward career goals. At the same time, most participants expressed low levels of turnover intention, suggesting a high level of staff retention within the study setting. Importantly, career growth was found to be strongly and inversely associated with turnover intention, indicating that nurses who perceived better career development opportunities were less likely to consider leaving their jobs. In contrast, sociodemographic characteristics did not show a significant influence on career growth. Taken together, these results highlight the central role of career growth in supporting staff retention among nurses in the studied hospital.

## 4. Discussion

The findings indicate a highly experienced, predominantly female, and multicultural nursing workforce, with generally positive perceptions of professional development and career progression, alongside low levels of turnover intention. Notably, turnover intention emerged as the only variable strongly associated with career growth, underscoring the pivotal role of career development opportunities in reducing nurses’ intentions to leave the organization.

Importantly, a strong and statistically significant negative correlation was observed between career growth and turnover intention (*r* = −0.718, *P* < .001), indicating that nurses who perceive greater career growth are substantially less likely to consider leaving their jobs. This finding aligns with previous empirical evidence identifying perceived career growth as a key protective factor against turnover intention among nurses.^[15,16]^ Clear pathways for advancement and opportunities for skill development enhance organizational commitment and workforce retention. These results are further supported by earlier studies demonstrating positive associations between organizational support, structured career development plans, and career growth.^[17]^ Additionally, while Malalasekara and Sivalogathasan reported a positive relationship between workload and job satisfaction^[18]^ and House et al found no general association between professional role or demographic factors and job satisfaction, the latter study noted that among military personnel, professional role, race/ethnicity, and education were significantly related to intent to stay.^[9]^ Collectively, these findings reinforce the importance of career development and organizational context in influencing nurses’ retention.

This result has significant applied importance; it showed the influence of the availability of development opportunities within the work environment on their decision to stay or leave. Nurses who see their career prospects within the organization as continually evolving, whether through training, recognition of competencies, or assignment to higher-level tasks, are more likely to remain in their jobs and find strong rationales for staying. This correlation reflected a close relationship between the employee’s perspective as per his/her career path and the extent of his/her job stability, when the nurse is granted growth and development opportunities and feels appreciated through the investment of the organization in his/her evolvement, he/she develops a belief that staying in this environment will provide them with a better future. Conversely, the absence of this growth or a state of professional stagnation generates feelings of frustration and boredom, making the idea of moving to another location seem more logical. Hence, the results showed how career growth becomes a decisive factor in determining whether healthcare workers will stay or leave.

In this study, the majority of nurses reported higher levels of emotional control, competence, communication skills, responsibility, and professional adaptability. These findings are consistent with recent studies suggesting that prolonged clinical exposure and organizational support enhance nurses’ emotional intelligence, resilience, and professional confidence.^[19-21]^ Nurses with more than ten years of experience, who constituted over half of the sample, are more likely to report higher self-efficacy and professional maturity, which may explain the positive perceptions observed.^[21]^ Comparable studies conducted in hospital settings have demonstrated that continuous professional development programs, mentoring, and supportive leadership significantly contribute to improvements in nurses’ professional attributes.^[22,23]^ The current findings suggest that the military hospital environment may provide structured systems that foster professional growth and role adaptation. Nurses in this study reported favorable perceptions of career promotion, salary growth, and professional prestige, with more than half strongly agreeing that their work achievements enhanced their professional status. These results align with prior research indicating that transparent promotion systems and recognition mechanisms are key drivers of career satisfaction and motivation among nurses.^[24]^

One of the most significant findings of this study is the low level of turnover intention among nurses, with the majority strongly disagreeing with statements related to leaving their job. This contrasts with several regional and international studies reporting moderate to high turnover intention among hospital nurses, particularly following the coronavirus pandemic-19 (COVID-19) pandemic.^[21,25]^ The relatively low turnover intention observed may be attributed to job security, structured career ladders, and perceived opportunities for growth within the military healthcare system. The multiple linear regression analysis further confirmed turnover intention as the only significant predictor of career growth, explaining a substantial proportion of variance in career growth perceptions. The absence of significant effects from demographic variables such as age, gender, education, nationality, and income suggests that organizational and psychological factors outweigh personal characteristics in shaping career growth perceptions. Therefore, it is recommended that periodic surveys be conducted to assess turnover intention, career growth, and related factors in order to evaluate the effectiveness of professional development programs using reliable scientific tools. The findings of these surveys should inform timely remedial actions and organizational improvements. In addition, improving working conditions in military hospitals by fostering a healthy work environment that promotes cooperation and teamwork, while reducing job-related pressures and professional burdens that contribute to burnout and turnover intention is essential.

Furthermore, sustainable and systematic professional development programs should be established for nurses, including training courses and specialized workshops addressing clinical, administrative, and leadership competencies. These programs should be aligned with the operational needs of military hospitals and the objectives of Saudi Vision 2030. Finally, nurse retention policies should emphasize continuous professional development, work–life balance, and attention to nurses’ psychological and social well-being, as these factors are critical for strengthening organizational commitment and long-term retention. Contrary to expectations, demographic characteristics were not significantly associated with career growth. This finding suggests that nurses’ perceptions of career growth may be influenced more strongly by organizational practices, such as opportunities for promotion, professional development, and managerial support, than by individual demographic characteristics. Similar findings have been reported in previous studies, indicating that organizational context plays a greater role in shaping career growth perceptions than personal characteristics.

This study has several limitations that should be considered when interpreting the findings. First, the cross-sectional design precludes the establishment of causal relationships between career growth and turnover intention. Although a significant association was observed, the direction of this relationship cannot be determined, and unmeasured factors may have influenced both variables. Second, the use of convenience sampling and participant recruitment through social media platforms such as WhatsApp may have introduced selection bias, as nurses who are more active on digital platforms or professional networks may have been more likely to participate. Consequently, the findings may not be fully representative of all nurses working in military hospitals or other healthcare settings in Saudi Arabia. Third, the study relied on self-reported questionnaires, which are susceptible to response biases, including social desirability and recall bias. Finally, because the study was conducted in a single military hospital, the generalizability of the findings to other healthcare institutions may be limited, as organizational culture, career development opportunities, and workplace policies can vary across settings. Future research should employ longitudinal or prospective study designs and consider advanced analytical approaches, such as structural equation modeling, to better examine the causal pathways and potential mediating or moderating mechanisms linking career growth, organizational factors, and turnover intention.

## 5. Conclusion

The findings reported that the nurses in the current study have positive perceptions of professional ability development, career promotion, prestige, and progress toward career goals, indicating a supportive professional environment within the military hospital setting. Importantly, nurses demonstrated low turnover intention, suggesting a high degree of job satisfaction and organizational commitment. In addition, turnover intention was strongly and negatively associated with perceived career growth. Nurses who experienced greater career growth were significantly less likely to express intentions to leave their jobs. This relationship was further confirmed through regression analysis, in which turnover intention emerged as the only significant predictor of career growth, explaining a substantial proportion of its variance. These findings underscore the central role of career growth opportunities in retaining nurses within military healthcare settings. Enhancing professional development, ensuring clear career pathways, and fostering a supportive work environment may be more influential in reducing turnover intention than demographic or structural factors alone. Overall, the study highlights the importance of strategic career development initiatives as a key mechanism for promoting nurse retention, workforce stability, and the sustained delivery of high-quality healthcare services in military hospitals.

## Acknowledgments

The authors of this study extend their appreciation to the Ongoing Research Funding Program (ORF-2026-1032), King Saud University, Riyadh, Saudi Arabia.

## Author contributions

**Conceptualization:** Amal Bany Ateah, Ahmad M. Rayani.

**Data curation:** Amal Bany Ateah, Ahmad M. Rayani.

**Formal analysis:** Amal Bany Ateah, Ahmad Aboshaiqah, Ahmad M. Rayani.

**Funding acquisition:** Amal Bany Ateah, Ahmad Aboshaiqah, Ahmad M. Rayani.

**Investigation:** Amal Bany Ateah, Ahmad M. Rayani.

**Methodology:** Ahmad M. Rayani.

**Project administration:** Ahmad Aboshaiqah, Ahmad M. Rayani.

**Resources:** Ahmad M. Rayani.

**Software:** Ahmad Aboshaiqah, Ahmad M. Rayani.

**Supervision:** Ahmad Aboshaiqah, Ahmad M. Rayani.

**Validation:** Amal Bany Ateah, Ahmad Aboshaiqah.

**Visualization:** Ahmad Aboshaiqah, Ahmad M. Rayani.

**Writing – original draft:** Amal Bany Ateah, Ahmad Aboshaiqah, Ahmad M. Rayani.

**Writing – review & editing:** Amal Bany Ateah, Ahmad Aboshaiqah, Ahmad M. Rayani.



## References

[1] Alshmemri M. The relationship between quality of life and nurses’ turnover intentions: a cross-sectional study. J Nurs Manag. 2025;2025:4951493. 40786707 10.1155/jonm/4951493 PMC12334292

[2] Nemati-Vakilabad R Kamalifar E Jamshidinia M Mirzaei A. Assessing the relationship between nursing process competency and work environment among clinical nurses: a cross-sectional correlational study. BMC Nurs. 2025;24:134. 39910530 10.1186/s12912-025-02760-3 PMC11800566

[3] Albalawi AM. Factors influencing nurses turnover in Saudi Arabia: a systematic review. in Nursing Forum, 2024, vol. 2024. Wiley Online Library:4987339.

[4] Sandvik H Hunskaar S. Staff retention and mortality. vol. 387, ed: British Medical Journal Publishing Group, 2024. 10.1136/bmj.q2521 39566970

[5] AbdELhay ES Taha SM El-Sayed MM Helaly SH AbdELhay IS. Nurses retention: the impact of transformational leadership, career growth, work well-being, and work-life Balance. BMC Nurs. 2025;24:148. 39923025 10.1186/s12912-025-02762-1 PMC11807322

[6] Alqahtani ON Alenazi MA Alanazi WI. Factors associated with nurses’ intention to leave in saudi arabia: a literature review. bjns. 2024;4:74–9.

[7] Saleh ZT Aslanoğlu A Elshatarat RA. Exploring the reasons and significant influencing factors of serious turnover intentions among nurses in Saudi Arabia. J Educ Health Promot. 2025;14:206. 40575523 10.4103/jehp.jehp_1227_24 PMC12200021

[8] Alshaibani NM Aboshaiqah AE Alanazi NH. Association of job satisfaction, intention to stay, organizational commitment, and general self-efficacy among clinical nurses in Riyadh, Saudi Arabia. Behav Sci. (Basel). 2024;14:1140. 39767281 10.3390/bs14121140 PMC11673037

[9] House S Crandell J Miller M Stucky C. The impact of professional role and demographic characteristics on job satisfaction and retention among healthcare professionals in a military hospital. Nurs Forum. 2022;57:1034–43. 35809050 10.1111/nuf.12777 PMC10083962

[10] Marufu TC Collins A Vargas L Gillespie L Almghairbi D. Factors influencing retention among hospital nurses: systematic review. Br J Nurs. 2021;30:302–8. 33733849 10.12968/bjon.2021.30.5.302

[11] Kaddourah B Abu-Shaheen AK Al-Tannir M. Quality of nursing work life and turnover intention among nurses of tertiary care hospitals in Riyadh: a cross-sectional survey. BMC Nurs. 2018;17:43. 30302056 10.1186/s12912-018-0312-0 PMC6167796

[12] Meyer VM Benjamens S Moumni ME Lange JFM Pol RA. Global overview of response rates in patient and health care professional surveys in surgery: a systematic review. Ann Surg. 2022;275:e75–81. 32649458 10.1097/SLA.0000000000004078 PMC8683255

[13] Alreshidi NM Alrashidi LM Alanazi AN Alshammri EH. Turnover among foreign nurses in Saudi Arabia. J Public Health Res. 2021;10:1971. 33849251 10.4081/jphr.2021.1971 PMC8054764

[14] Ni YX Li L Li JP. Development and psychometric evaluation of the career growth scale for nurses. Asian Nurs Res (Korean Soc Nurs Sci). 2023;17:200–7. 37652261 10.1016/j.anr.2023.08.001

[15] Al Balushi AK Thumiki VRR Nawaz N Jurcic A Gajenderan V. Role of organizational commitment in career growth and turnover intention in public sector of Oman. PLoS One. 2022;17:e0265535. 35551528 10.1371/journal.pone.0265535 PMC9098061

[16] Nashwan AJ. The vital role of career pathways in nursing: a key to growth and retention. Cureus. 2023;15:e38834. 37303402 10.7759/cureus.38834 PMC10254089

[17] Abdel Wahab EA El-Sayed RI. Factors influencing career development among nursing staff at port-said governmental hospitals. Port Said Scientific J Nurs. 2020;7:191–211.

[18] Malalasekara L Sivalogathasan V. Factors influencing job satisfaction of nurses attached to national institute of nephrology, dialysis and transplant. Manag Issues. 2020;5:14.

[19] Chatzidimitriou E Triantari S Zervas I. Emotional intelligence in the professional development of nurses: from training to the improvement of healthcare quality. Nurs Rep. 2025;15:275. 40863663 10.3390/nursrep15080275 PMC12389285

[20] Turjuman F Alilyyani B. Emotional intelligence among nurses and its relationship with their performance and work engagement: a cross-sectional study. J Nurs Manag. 2023;2023:5543299. 40225659 10.1155/2023/5543299 PMC11919067

[21] Labrague LJ. Resilience as a mediator in the relationship between stress-associated with the Covid-19 pandemic, life satisfaction, and psychological well-being in student nurses: a cross-sectional study. Nurse Educ Pract. 2021;56:103182. 34508944 10.1016/j.nepr.2021.103182 PMC8425956

[22] Boamah SA Laschinger HKS Wong C Clarke S. Effect of transformational leadership on job satisfaction and patient safety outcomes. Nurs Outlook. 2018;66:180–9. 29174629 10.1016/j.outlook.2017.10.004

[23] Lysfjord EM Skarstein S. Empowering leadership: a journey of growth and insight through a mentoring program for nurses in leadership positions. J Healthc Leadersh. 2024;16:443–54. 39502081 10.2147/JHL.S482087 PMC11537044

[24] Ghanem Atalla AD Sharif LS Katooa NE Kandil FS Mahsoon A Mahmoud Elseesy NA. Relationship between nurses’ perception of professional shared governance and their career motivation: a cross-sectional study. Int J Nurs Sci. 2023;10:485–91. 38020835 10.1016/j.ijnss.2023.09.016 PMC10667319

[25] Albougami AS Almazan JU Cruz JP. Factors affecting nurses’ intention to leave their current jobs in Saudi Arabia. Int J Health Sci (Qassim). 2020;14:33–40. PMC7269627 32536847

