# Supplementary material for: Sociodemographic factors and career growth as determinants of staff retention among nurses in a military hospital in Saudi Arabia
Source: Medicine (Baltimore). 2026 Aug 28;105(35):e50431. doi: 10.1097/MD.0000000000050431 (PMC13529174; doi:10.1097/MD.0000000000050431)
Supplement: Supplementary file 1 [file medi-105-e50431-s001.doc]

**Supplementary** Table 1 multiple linear regression analysis of predictors of career growth among nurses

| **Variables** | **Unstandardized Coefficients** | | **Standardized Coefficients**  **Beta** | **t** | **Sig** | **95.0% Confidence Interval for B** | |
| --- | --- | --- | --- | --- | --- | --- | --- |
|  |  |  |  |  |  | **Lower Bound** | **Upper Bound** |
|  | **B** | **Std. Error** |  |  |  |  |  |
| (Constant) | 105.406 | 6.557 |  | 16.075 | <.001 | 92.508 | 118.304 |
| Age | -.066 | 1.419 | -.003 | -.047 | .963 | -2.857 | 2.725 |
| Gender | 1.206 | 2.473 | .019 | .487 | .626 | -3.659 | 6.070 |
| Years of Experience | -1.047 | .951 | -.059 | -1.101 | .272 | -2.918 | .823 |
| Department | .086 | .278 | .012 | .310 | .757 | -.461 | .634 |
| Education | -.156 | 1.334 | -.004 | -.117 | .907 | -2.780 | 2.469 |
| Monthly income | .140 | .996 | .006 | .140 | .888 | -1.820 | 2.099 |
| Nationality | .679 | .904 | .034 | .751 | .453 | -1.100 | 2.457 |
| Turnover intention | -3.609 | .191 | -.715 | -18.898 | <.001 | -3.985 | -3.234 |
